# Serum Uric Acid Is Associated with the Progression of Left Ventricular Diastolic Dysfunction in Apparently Healthy Subjects

**DOI:** 10.1155/2022/9927254

**Published:** 2022-10-15

**Authors:** Chen Die Yang, Shuo Feng, Jia Wei Chen, Muladili Aihemaiti, Xin Yi Shu, Jin Wei Quan, Feng Hua Ding, Lin Lu, Wei Feng Shen, Rui Yan Zhang, Xiao Qun Wang

**Affiliations:** ^1^Department of Cardiovascular Medicine, Ruijin Hospital, Shanghai Jiao Tong University School of Medicine, Shanghai, China; ^2^Institute of Cardiovascular Disease, Shanghai Jiao Tong University School of Medicine, Shanghai, China

## Abstract

**Background:**

Left ventricular (LV) diastolic dysfunction (LVDD) is the defining feature of heart failure with preserved ejection fraction (HFpEF) and predicts subsequent incident heart failure (HF) and all-cause mortality. Mounting evidence reveals that cardiometabolic risk factors play critical roles in the development of LVDD. In this study, we sought to investigate the relation between serum uric acid (SUA) level and the progression of LVDD in apparently healthy patients.

**Methods:**

A total of 1082 apparently healthy subjects without diagnosed cardiovascular disease and LVDD were consecutively enrolled. SUA levels were measured, and repeat echocardiography and tissue Doppler imaging (TDI) were performed at baseline and during 1-year follow-up.

**Results:**

By dividing the study population based on quartiles of SUA, we found subjects in higher quartiles had greater increases in TDI-derived early diastolic velocity (e′) and E (peak LV filling velocity)/e′ ratios during 1-year follow-up. After multivariate adjustment, high SUA persisted to be an independent predictor for the subsequent worsening of LVDD (odds ratio: 1.351 [95% CI 1.125~1.625], per 100 *μ*mol/L SUA). Subgroup analysis suggested that the association between SUA and LVDD development was more pronounced in subjects without other cardiometabolic risk factors involved. Factor analysis demonstrated that high SUA was the major cardiometabolic attribute in patients with LVDD progression.

**Conclusion:**

Our findings suggest that high SUA is an independent cardiometabolic risk factor for the progression of LVDD in apparently healthy subjects.

## 1. Background

Left ventricular (LV) diastolic dysfunction (LVDD) is recognized as the hallmark of heart failure (HF) with preserved ejection fraction (EF, HEpEF) that has different clinical features but similar poor prognosis as compared to those with reduced EF [1–4]. With comprehensive echocardiography and tissue Doppler imaging (TDI) examination, LVDD is found to be prevalent in 11.1%~34.7% of the general population [5–9], varying according to different criteria used, and is usually presented without recognized symptoms of HF [5, 6]. Mounting evidence reveals that LVDD is associated with the subsequent development of HF and all-cause mortality [6, 10, 11]. Especially, patients with metabolic disturbance, including diabetes, obesity, and metabolic syndrome (MetS), suffer profoundly higher risk of LVDD than those without cardiometabolic risk factors [12–16].

Serum uric acid (SUA) is a metabolic end-product of purine metabolism by xanthine oxidase (XO). Emerging data reveal that SUA is closely related to cardiovascular risk and events, such as hypertension, coronary artery disease, and HF [17]. Moreover, SUA-lowering therapy by XO inhibitor displays benefits in improving cardiovascular outcomes both in clinical and animal studies [18–21]. Previously, several echocardiographic surveys demonstrated a positive relationship between SUA and markers of LVDD [22–24]. However, current data on the relationship between SUA and LVDD were almost obtained from cross-sectional surveys. Little is known regarding the impact of SUA on longitudinal changes of LVDD.

In the present study, we analyzed the relation between SUA and LVDD progression in apparently healthy patients by repeat echocardiography and TDI assessment during 1-year follow-up.

## 2. Methods

### 2.1. Study Design

**Figure 1:**
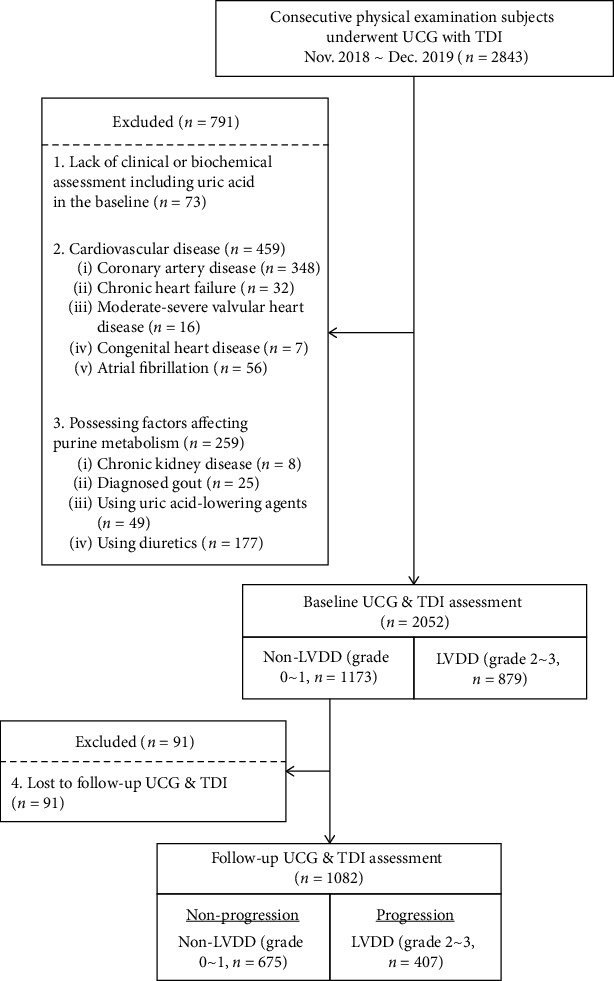
Flow chart of patient enrollment. LVDD: left ventricular diastolic dysfunction; TDI: tissue Doppler imaging; UCG: ultrasound cardiogram.

A total of 2,843 subjects between 18 to 80 years old who had physical examination including echocardiography and TDI in Ruijin Hospital, Shanghai Jiao Tong University School of Medicine from November 2018 to December 2019 were consecutively enrolled in the study. Exclusion criteria were as follows: (1) lack of clinical or biochemical assessment including SUA in the baseline (*n* = 73); (2) history of diagnosed cardiovascular disease (*n* = 459), which includes coronary artery disease (*n* = 348), chronic HF (*n* = 32), moderate to severe valvular heart disease (*n* = 16), congenital heart disease (*n* = 7), and atrial fibrillation (*n* = 56); (3) possessing factors that affect purine metabolism (*n* = 259), which include chronic kidney disease (*n* = 8), diagnosed gout (*n* = 25), medication use of uric-acid lowering agents (*n* = 49), and diuretics (*n* = 177). Thus, a total of 2052 subjects were enrolled in the baseline and their LVDD was graded by TDI. There were 1173 subjects with normal LV diastolic function (grade 0) or mild LVDD (grade 1) and 879 subjects with moderate (grade 2) or severe (grade 3) LVDD. Since this study is aimed at analyzing LVDD progression, we only selected subjects with non-LVDD (grade 0~1) in the cohort. These subjects were then followed-up for around 12 months and underwent repeated echocardiography and TDI assessment. After exclusion of 91 subjects who lost to follow-up, the remaining 1082 subjects comprised the final analysis **(**Figure 1**)**.

### 2.2. Clinical and Biochemical Assessments

Detailed information of medical history and lifestyles were obtained using a standard questionnaire by trained physicians. Body mass index (BMI) was calculated as weight/height^2^ (kilograms per square meter). Body surface area (BSA) was calculated as 0.0061 × height + 0.0128 × weight − 0.1529. Blood pressure was measured on the nondominant arm in seated position after a 10-minute rest. Three measurements were taken at 1-minute interval, and the average was used for analysis. The diagnosis of type 2 diabetes was made according to the criteria of American Diabetes Association (symptoms of diabetes with casual plasma glucose concentration ≥ 200 mg/dL [11.1 mmol/L] or fasting plasma glucose ≥ 126 mg/dL [7.0 mmol/L], 2 h postprandial glucose ≥ 200 mg/dL [11.1 mmol/L] during an oral glucose tolerance test (OGTT) and currently or previously treated with insulin and/or oral hypoglycemic agents) [25]. Hypertension was diagnosed according to seventh report of the Joint National Committee on prevention, detection, evaluation, and treatment of high blood pressure (JNC 7) [26].

All the blood samples, except for postprandial testing, were drawn after an overnight fasting. OGTT was performed with 75 g glucose, and blood was collected for the measurement of postprandial glucose and insulin after 2 h. SUA, fasting and postprandial plasma glucose, insulin, creatinine, alanine aminotransferase (ALT), aspartate aminotransferase (AST), total cholesterol, low-density lipoprotein (LDL) cholesterol, high-density lipoprotein (HDL) cholesterol, and triglycerides were assessed (HITACHI 912 Analyzer, Roche Diagnostics, Germany). Blood HbA1c was measured using ion-exchange high performance liquid chromatography with Bio-Rad Variant Hemoglobin Testing System (Bio-Rad Laboratories, USA). Serum levels of high sensitive C-reactive protein (hsCRP) were determined by ELISA (Biocheck Laboratories, Toledo, OH, USA). Estimated glomerular filtration rate (eGFR) was computed using the Chronic Kidney Disease Epidemiology Collaboration equation. Homeostasis model assessment-estimated insulin resistance (HOMA-IR) was calculated according to the formula as follows: fasting insulin (*μ*U/L) × fasting glucose (mmol/L)/22.5 [27].

### 2.3. Echocardiographic Examination

Transthoracic echocardiography was performed using a commercially available system (Vivid-I, GE Healthcare, Milwaukee, WI) with a 1.9 to 3.8 mHz phased-array transducer. All data were stored digitally, and offline data analysis was performed (EchoPac, version 7; GE Healthcare).

EF was calculated using the modified Simpson's biplane technique. The LV length was measured in the apical 4-chamber view. To facilitate application of clinical normality cut points, LV end-diastolic volume (LVEDV), LV end-systolic volume (LVESV), and LV mass were indexed by BSA calculated at each study time point. LV mass was estimated from M-mode measurements by the formula LV mass = 0.8 × 1.04 × [(LVEDD + IVST + LVPWT)^3^ − LVEDD^3^] + 0.6, where LVEDD is LV end-diastolic diameter, IVST is interventricular septal thickness, and LVPWT is LV posterior wall thickness.

Transmitral inflow was recorded using pulsed wave Doppler in the apical 4-chamber view for measurements of early (E) and late (A) mitral inflow velocities. Early diastolic velocity was assessed at the septal (septal e′) and lateral (e′) sites of the mitral annulus using pulsed-wave TDI. Mean E/e′ ratio was obtained by averaging the septal and lateral mitral annulus to estimate LV filling pressure. Patients were classified into four groups according to diastolic function based on EACVI/ASE recommendations [28] as follows: normal diastolic function (grade 0), mild (grade 1), moderate (grade 2) or severe (grade 3) LVDD. Patients with both septal e′ ≥ 8 cm/s and lateral e′ > 10 cm/s constituted the group in grade 0. For the remaining patients, the following classification was used: grade 1 when E/A < 0.8, mean E/e′ ≤ 8; grade 2 when E/A was between 0.8 and 2, mean E/e′ between 8 and 13; and grade 3 when E/A > 2, mean E/e′ ≥ 13. In this study, development of LVDD from grade 0~1 to grade 2~3 was defined as LVDD progression.

### 2.4. Statistical Analyses

Continuous variables were presented as median (interquartile range [IQR]) or mean ± SD, and categorical data were summarized as frequencies (percentages). Normal distribution of continuous variables was evaluated by Shapiro-Wilk test. For normally distributed variables, differences in quartiles of SUA and subgroup analysis were performed by one-way analysis of variance (ANOVA) followed by post hoc *t*-test with Bonferroni correction. For nonnormally distributed continuous variables, differences were analyzed by the Mann–Whitney *U* test or Kruskal-Wallis test. Differences in categorical variables were analyzed by *χ*2 test. Correlation between SUA and mean E/e′ ratio was determined by Spearman's correlation test. Univariate logistic regression analysis was performed to identify univariate determinants of LVDD progression. Afterwards, multivariate regression was performed by entering all the conventional risk factors and significant determinates in the univariate analysis after backward elimination. SUA was analyzed both as continuous and categorical variables in univariate and multivariate models. All statistical analyses were performed using the R statistical package v.4.0.3 (R Project for Statistical Computing, Vienna, Austria). A 2 − tailed < 0.05 was considered statistically significant.

For the exploratory factor analysis, principal component analysis (PCA) was performed to reduce intercorrelated variables to fewer clustering factors that retain as the much of variance in the original variables as possible. Higher factor loadings denote higher correlation between the given variable and the clustering factor. Bartlett's test of sphericity was implemented to test whether a correlation matrix is different from an identity matrix and support the need for data reduction. Z scores were calculated to scale all the variables to standard scores before PCA. An eigenvalue > 1 was used as the extraction method and varimax rotation. We included only the highest ranked variables with at least shared variance between the variable and clustering factors (factor loading ≥ 15%) in interpreting factors.

## 3. Results

### 3.1. Baseline Characteristics of the Cohort

A total of 1082 subjects were enrolled in the cohort and were followed-up for 12.5 ± 1.5 months. The mean age was 51.0 ± 11.2 years and the male-to-female ratio was 63.5% to 36.5%. There were 8.3% subjects with hypertension and 15.1% with type 2 diabetes. The mean level of SUA was 341.2 ± 82.9 *μ*mol/L. Of note, male subjects had markedly higher SUA level as compared to that of females (374.5 ± 74.6 vs. 283.2 ± 62.2 *μ*mol/L, *P* < 0.001).

We divided the entire population of the cohort into quartiles based on SUA levels (SUA ≤ 282 *μ*mol/L; 283~340 *μ*mol/L; 341~393 *μ*mol/L; ≥ 394 *μ*mol/L; Table 1). We found subjects with higher quartiles of SUA were more frequently to be male, having smoking habits, higher levels of BMI, systolic and diastolic blood pressure, fasting glucose, lipid, hsCRP, and insulin resistance levels, but poorer hepatic and renal function. There was no significant difference in history of hypertension and diabetes and medical treatments among the 4 groups.

### 3.2. Geometric and Functional Echocardiographic Analyses

**Figure 2:**
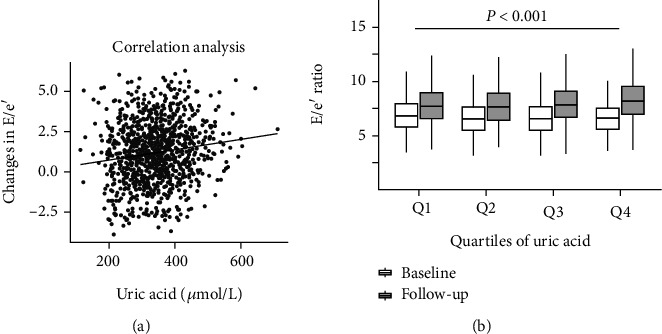
Relation of serum uric acid and changes in E/e′ ratio during follow-up. (a) Correlation between changes in E/e′ ratio and serum uric acid. (b) Changes in E/e′ ratio in different quartiles of serum uric acid (Q1: ≤282 *μ*mol/L; Q2: 283~340 *μ*mol/L; Q3: 341~393 *μ*mol/L; Q4: ≥394 *μ*mol/L) were tested by one-way ANOVA (*P* < 0.001). Horizontal lines in the box: upper, 75% percentile; middle, median; lower, 25% percentile.

After a 12-month follow-up, 37.6% of subjects in the cohort progressed to moderate to serve LVDD (grade 2~3). The mean E/e′ ratios were 6.75 ± 1.55 cm/s at baseline and 7.93 ± 1.93 cm/s at the 12-month follow-up. The mean change in E/e′ ratio was 1.18 ± 1.85 cm/s. There was a positive correlation between baseline SUA level and changes in E/e′ ratio during follow-up (Spearman's *r* = 0.140, *P* < 0.001; Figure 2(a)). Changes in E/e′ ratio during follow-up were also increased across quartiles of SUA (*P* < 0.001; Figure 2(b)).

Geometric and functional echocardiographic parameters were compared between patients in different quartiles of SUA (Table 2). We found left atrial diameter, diastolic and systolic LV diameters, LV wall thickness, and mass index were stepwise increased with increasing quartiles of SUA both at baseline and during follow-up. However, changes in these LV geometric parameters, with the exception for LVEDD, were comparable between the 4 groups. No significant changes in EF were detected.

In the baseline, e′ and E/e′ ratios were similar in different quartiles of SUA both at septal and lateral sides of mitral annulus. However, increases in E/e′ at both sides and the averaged values were markedly elevated in subjects in higher quartiles, whereas peak atrial filling velocity (A) and E/A ratio as well as their respective changes were comparable among different quartiles.

### 3.3. Univariate and Multivariate Analyses

Univariate analyses (Table 3) revealed that predictors for LVDD progression were older age (OR: 2.466 [95% CI 2.138~2.864], per 10 years), male sex (OR: 1.908 [95% CI 1.462~2.503]), higher systolic (OR: 1.232 [95% CI 1.122~1.357], per 10 mmHg) and diastolic blood pressure (OR: 1.221 [95% CI 1.063~1.406], per 10 mmHg), the presence of hypertension (OR: 2.638 [95% CI 1.581~4.431]) and diabetes (OR: 2.595 [95% CI 1.852~3.648]), current smokers (OR: 1.461 [95% CI 1.067~2.000]), higher levels of HbA1c (OR: 1.688 [95% CI 1.380~2.088]), fasting glucose (OR: 1.230 [95% CI 1.105~1.381]), triglyceride (OR: 1.436 [95% CI 1.255~1.659]), medication use of beta blocker (OR: 1.889 [95% CI 1.114~3.208]), renin-angiotensin-aldosterone system inhibitor (OR: 2.403 [95% CI 1.380~4.239]), calcium channel blocker (OR: 3.038 [95% CI 1.489~6.465]), and statin (OR: 2.997 [95% CI 1.872~4.869]). Meanwhile, lower HDL cholesterol (OR: 0.361 [95% CI 0.243~0.530]), poorer renal (eGFR, OR: 0.750 [95% CI 0.685~0.818]), and LV function (LVEF, OR: 0.942 [95% CI 0.909~0.976]) were also associated with LVDD progression. When treated as categorical variables, quartiles of SUA were positively associated with progression of LVDD (*P* for trend <0.001). SUA ≥ 394 *μ*mol/L corresponded to a 2.697-fold [95% CI 1.892~3.867] increased risk for progression of LVDD as compared to SUA ≤ 282 *μ*mol/L. Similarly, when treated as a continuous variable, SUA level remained positively associated with LVDD progression (OR: 1.528 [95% CI 1.311~1.786], per 100 *μ*mol/L).

**Figure 3:**
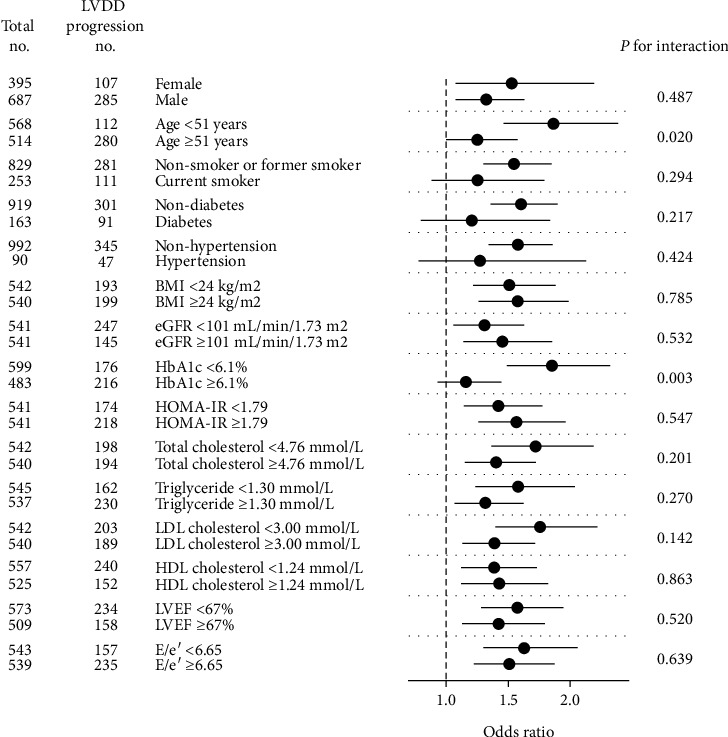
Subgroup analysis by forest plot. Forest plot shows the association between serum uric acid level and LVDD progression in different subgroups and the significance of the corresponding interaction terms. The dashed reference line indicates odds ratio of 1.0. The number of all the patients and the number of patients with LVDD progression in each subgroup are labelled. BMI: body mass index; eGFR: estimated glomerular filtration rate; HbA1c: glycated hemoglobin A1c; HDL: high-density lipoprotein; HOMA-IR: homeostasis model assessment-estimated insulin resistance; LDL: low-density lipoprotein; LVDD: left ventricular diastolic dysfunction; LVEF: left ventricular ejection fraction.

Multivariate analyses (Table 3) were performed by entering all the conventional risk factors and significant predictors from the univariate analyses followed by stepwise backward elimination. Age, diastolic blood pressure, the presence of diabetes, triglyceride, EF, and SUA remained in the model with significant association with LVDD progression. After multivariate adjustment, SUA ≥ 394 *μ*mol/L corresponded to a 1.851-fold [95% CI 1.215~2.828] increased risk for progression of LVDD as compared to SUA ≤ 282 *μ*mol/L. Every 100 *μ*mol/L increase in SUA conferred a 1.351-fold [95% CI 1.125~1.625] higher risk of LVDD progression when treated as a continuous variable. Furthermore, subgroup analyses (Figure 3) demonstrated that patients with higher SUA developed LVDD progression regardless of sex, BMI, renal function, lipid levels, and LV systolic and diastolic function at baseline, whereas the association was only present in subgroups who were of younger age (<51 years), with lower HbA1c (<6.1%) or without hypertension, diabetes, and smoking habits. There were also significant interaction terms between age, HbA1c, and SUA on LVDD progression in the bivariate analysis of the overall population.

### 3.4. Factor Analysis

There was high degree of intercorrelation between various metabolic variables (Supplementary Table I). Especially, SUA was significantly associated with most of these metabolic variables except for postprandial glucose and total cholesterol. Therefore, factor analysis was performed to extract key uncorrelated metabolic factors in subjects with LVDD progression. Bartlett's test of sphericity was highly significant (*P* < 0.0001), indicating good model acceptability.

**Figure 4:**
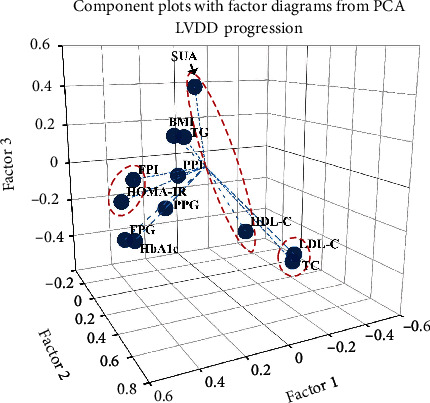
Factor analysis of metabolic patterns in association with LVDD progression. BMI: body mass index; FPG: fasting plasma glucose; FPI: fasting plasma insulin; HbA1c: glycated hemoglobin; HDL-c: high-density lipoprotein cholesterol; HOMA-IR: homeostatic model assessment of insulin resistance; LDL-c: low-density lipoprotein cholesterol; LVDD: left ventricular diastolic dysfunction; PCA: principal component analysis; PPG: postprandial plasma glucose (2 hours); PPI: postprandial plasma insulin (2 hours); TC: total cholesterol; TG: triglyceride; SUA: serum uric acid.

We identified an insulin resistance factor as the initial factor that accounted for 27.9% of the variance, a second cholesterol factor accounted for 19.0% of the variance, and a third factor comprised of SUA as a major component accounted for 14.7% of the variance. Taken together, these factors accounted for 61.5% of the total variance in measured variables (Supplementary Table II and Figure 4).

## 4. Discussion

The major findings of the present study are that SUA levels are positively related to increase in E/e′ in apparently healthy subjects with non-LVDD (grade 0~1). SUA is an independent predictor for the progression of LVDD after multivariate adjustment of conventional risk factors.

Previous population-based studies revealed that LVDD is prevalent in the general population [5–9] and is progressed rapidly over time. A large-scale community-based study in Minnesota showed that the prevalence of LVDD increased from 23.8% to 39.2% by repeat echocardiography examinations after 4 years [10]. In a retrospective study of outpatient patients, LVDD was present in 72.3% of patients, and 16% had worsening diastolic function after 1 year [29]. Consistent with these findings, 42.8% of the subjects in the present study were with LVDD. During 1-year follow-up, 37.6% of the remaining subjects with non-LVDD developed worse diastolic dysfunction. These data support the concept that LVDD is rapidly evolved even in apparently healthy patients without obvious LVDD. Given that LVDD is the defining feature of HFpEF and an independent predictor for subsequent HF and mortality, early risk stratification and proper management of LVDD are warranted.

Several risk factors have been established for LVDD including age [30, 31], diabetes [12, 13], obesity [16], hypertension [32, 33], and LV hypertrophy [34]. Several lines of evidence suggest that SUA is also associated with LVDD in a variety of clinical conditions. SUA was shown to be associated with LVDD in apparently healthy patients with essential hypertension [22]. Elevated SUA was independently associated with the presence of LVDD criteria as septal e′velocity < 7 in military individuals [35]. In patients with dilated cardiomyopathy, Cicoira et al. found that there was a positive correlation between SUA level and mitral E wave velocity, E/A ratio, E wave deceleration time (DtE), and restrictive mitral filling pattern (RMFP) [23]. In a large-scale community-based research performed in asymptomatic Asians, hyperuricemia was closely linked to indices of LVDD and SUA set at 7.0 mg/dl provided the optimal cut-off to identify LVDD [24]. However, current data on the relationship between SUA and LVDD were merely based on cross-sectional surveys. Since LVDD is in essence a rapidly progressed LV functional abnormality, the role of SUA in the development of LVDD is still unclear.

In the present study, for the first time we reported that SUA level was not only related to impaired LVDD but also the subsequent development of LVDD over time. First, in apparently healthy subjects, those with higher SUA level tended to have worse LVDD. Second, SUA level was positively correlated to changes in E/e′ ratio during 1-year follow-up. After multivariate adjustment, SUA remained an independent predictor for the subsequent worsening of LVDD. Third, due to the fact that cardiometabolic variables were intercorrelated, factor analysis showed that SUA comprised the major cardiometabolic factors in patients with LVDD progression. Taken together, these findings demonstrated that SUA is an important cardiometabolic player, or at least a sensitive biomarker, in the development of LVDD. Interestingly, subgroup analysis showed that the association between SUA and LVDD progression was more pronounced in subjects with younger age, lower HbA1c, or those without hypertension, diabetes, and smoking habits, implying that the potential contribution of SUA to LVDD progression is greater when other conventional risk factors of LVDD are not involved. Therefore, there might be common downstream pathways underlying the development of LVDD in the setting of metabolic disturbance. Multiple cardiometabolic risk factors, as usually seen in the context of MetS, may have overlapping effects on LVDD progression.

Although it is still unclear whether SUA plays a causal role or just acts as a biomarker in LVDD progression, hyperuricemia is generally considered to be associated with increased XO activity in purine metabolism, which presumably promotes excess production of reactive oxygen species (ROS), thereby leading to reduced nitric oxide bioavailability, inflammatory state, endothelial dysfunction, myocardial fibrosis, and finally LVDD [18–21]. Actually, existing clinical and basic research evidence showed that treatment by XO inhibitor which lowered XO activity and thus SUA level would improve LVDD and clinical outcomes [36–39]. Nevertheless, the specific role of SUA in the development of LVDD and the precise mechanisms await precise characterization in future studies.

Our findings should be interpreted in the context of following limitations: first, this study is a retrospective analysis based on prospectively collected data, and all the enrolled patients were from a single center. Second, some LVDD parameters such as deceleration time and isovolumic relaxation time were not assessed. Moreover, combined use of different echocardiography measurements including stress echocardiography, Valsalva maneuver, and color M-mode flow propagation velocity in addition to TDI that we performed may provide more precise information.

## 5. Conclusions

In conclusion, our findings suggest that elevated SUA is independently associated with LVDD progression in apparently healthy subjects. Tight control of SUA by lifestyle intervention or medication optimization may provide favorable effects on the development of LVDD.

## Figures and Tables

**Table 1:** Baseline characteristics grouped by quartiles of serum uric acid.

SUA (*μ*mol/L)	≤282	283~ 340	341~ 393	≥394	*P* value
n	272	269	273	268	
Follow-up time, months	12.52 ± 1.57	12.46 ± 1.53	12.42 ± 1.46	12.63 ± 1.40	0.400
Male sex	68 (25.0)	138 (51.3)	230 (84.2)	251 (93.7)	<0.001
Age, years	48.59 ± 11.16	52.60 ± 11.32	51.60 ± 11.52	51.38 ± 10.32	<0.001
Hypertension	23 (8.5)	25 (9.3)	23 (8.4)	19 (7.1)	0.831
Diabetes	33 (12.1)	41 (15.2)	45 (16.5)	44 (16.4)	0.449
Smoking status	23 (8.5)	53 (19.7)	84 (30.8)	93 (34.7)	<0.001
BMI, kg/m^2^	22.51 ± 3.11	23.84 ± 3.07	24.65 ± 2.76	25.32 ± 3.51	<0.001
Systolic BP, mmHg	121.27 ± 15.92	124.00 ± 15.38	125.78 ± 15.34	127.22 ± 17.22	0.004
Diastolic BP, mmHg	71.31 ± 10.90	74.18 ± 9.87	74.77 ± 11.15	76.16 ± 10.66	<0.001
HbA1c, %	5.59 ± 0.53	5.74 ± 0.75	5.79 ± 0.75	5.77 ± 0.59	0.003
Fasting glucose, mmol/L	4.88 (4.53~5.34)	5.00 (4.61~5.51)	5.09 (4.70~5.59)	5.10 (4.70~5.59)	0.002
Postprandial glucose, mmol/L	6.64 (5.66~7.74)	7.06 (5.70~8.73)	6.92 (5.84~8.62)	7.03 (6.03~8.30)	0.169
Fasting insulin, *μ*U/L	6.33 (4.68~9.05)	7.46 (4.95~10.72)	8.46 (5.65~12.42)	8.93 (6.05~13.04)	<0.001
Postprandial insulin, *μ*U/L	36.37 (22.49~59.03)	43.54 (24.48~64.26)	44.22 (26.12~76.03)	44.98 (28.20~78.83)	0.028
HOMA-IR	1.41 (0.96~2.06)	1.73 (1.06~2.52)	2.00 (1.28~2.98)	2.06 (1.38~ 3.07)	<0.001
Alaine aminotransferase, IU/L	20.22 ± 15.99	23.06 ± 11.52	26.61 ± 14.54	29.82 ± 17.71	<0.001
Aspartate aminotransferase, IU/L	21.65 ± 20.79	20.85 ± 6.33	22.19 ± 8.26	23.27 ± 8.38	0.143
Triglyceride, mmol/L	1.04 (0.78~1.35)	1.18 (0.89~1.68)	1.44 (1.06~1.93)	1.75 (1.23~2.52)	<0.001
Total cholesterol, mmol/L	4.80 ± 0.97	4.79 ± 1.07	4.78 ± 1.04	4.95 ± 1.07	0.179
HDL cholesterol, mmol/L	1.49 ± 0.38	1.36 ± 0.36	1.21 ± 0.29	1.12 ± 0.26	<0.001
LDL cholesterol, mmol/L	2.92 ± 0.84	2.97 ± 0.91	3.02 ± 0.89	3.12 ± 0.89	0.052
Serum creatine *μ*mol/L	67.32 ± 47.88	72.08 ± 14.71	82.01 ± 49.50	85.59 ± 13.64	<0.001
Blood urea nitrogen, mmol/L	4.93 ± 1.47	5.31 ± 1.39	5.40 ± 1.68	5.60 ± 1.34	<0.001
eGFR, mL/min/1.732m^2^	111.76 ± 19.37	103.93 ± 15.81	10ll1.77 ± 14.40	98.45 ± 12.58	<0.001
hsCRP, mg/L	0.39 (0.21~1.18)	0.65 (0.29~1.17)	0.99 (0.42~2.25)	1.62 (0.65~3.64)	<0.001
Aspirin	15 (5.5)	20 (7.4)	16 (5.9)	11 (4.1)	0.425
P2Y_12_ inhibitor	21 (7.7)	16 (5.9)	12 (4.4)	10 (3.7)	0.174
Beta blocker	16 (5.9)	19 (7.1)	16 (5.9)	8 (3.0)	0.195
ACEI/ARB	13 (4.8)	13 (4.8)	15 (5.5)	12 (4.5)	0.956
Calcium channel blocker	12 (4.4)	6 (2.2)	7 (2.6)	7 (2.6)	0.430
Statin	13 (4.8)	26 (9.7)	22 (8.1)	16 (6.0)	0.122
OHA	10 (3.7)	17 (6.3)	12 (4.4)	10 (3.7)	0.414
Insulin	4 (1.5)	6 (2.2)	2 (0.7)	3 (1.1)	0.492

Data are expressed as mean ± standard deviation or median (interquartile range). ACEI: angiotensin-converting enzyme inhibitor; ARB: angiotensin receptor blocker; BMI: body mass index; BP: blood pressure; eGFR: estimated glomerular filtration rate; HbA1c: glycated hemoglobin A1c; HDL: high-density lipoprotein; HOMA-IR: homeostasis model assessment-estimated insulin resistance; hsCRP: high-sensitivity C-reactive protein; LDL: low-density lipoprotein; OHA: oral hypoglycemic agent; SUA: serum uric acid.

**Table 2:** Changes in echocardiography parameters during follow-up grouped by quartiles of serum uric acid.

SUA (*μ*mol/L)		≤282	283~340	341~393	≥394	*P* value
*n*		272	269	273	268	
LA, mm	B	34.55 ± 3.30	36.42 ± 3.05^‡^	36.95 ± 3.20^‡^	37.82 ± 3.06^‡^	<0.001
	F	34.50 ± 3.41	36.45 ± 3.20^‡^	37.03 ± 3.13^‡^	38.01 ± 3.04^‡^	<0.001
	Δ	−0.05 ± 2.38	0.03 ± 2.03	0.08 ± 1.88	0.19 ± 1.90	0.580

LVESD, mm	B	28.41 ± 2.36	29.57 ± 2.47^‡^	30.24 ± 2.60^‡^	30.81 ± 2.13^‡^	<0.001
	F	28.60 ± 2.25	29.62 ± 2.50^‡^	30.43 ± 2.45^‡^	30.91 ± 2.46^‡^	<0.001
	Δ	0.19 ± 2.04	0.05 ± 2.02	0.19 ± 1.85	0.10 ± 1.85	0.792

LVEDD, mm	B	45.84 ± 3.25	47.62 ± 3.39^‡^	48.51 ± 3.47^‡^	49.13 ± 2.93^‡^	<0.001
	F	45.64 ± 3.90	47.33 ± 3.50^‡^	48.76 ± 3.39^‡^	49.36 ± 3.06^‡^	<0.001
	Δ	−0.21 ± 3.28	−0.29 ± 2.15	0.25 ± 2.04	0.23 ± 2.02	0.012

LVESV index, mL/m^2^	B	19.38 ± 3.76	20.29 ± 3.88	20.12 ± 3.94	20.29 ± 3.46	0.070
	F	19.78 ± 3.41	20.11 ± 3.84	20.67 ± 3.98	20.77 ± 3.95	0.044
	Δ	0.40 ± 3.79	−0.19 ± 3.65	0.55 ± 3.76	0.47 ± 3.36	0.181

LVEDV index, mL/m^2^	B	61.46 ± 9.21	62.97 ± 8.74	62.32 ± 9.47	62.43 ± 8.09	0.450
	F	60.95 ± 8.11	61.87 ± 9.16	62.33 ± 8.85	62.74 ± 8.42	0.244
	Δ	−0.52 ± 7.69	−1.10 ± 7.46	0.00 ± 6.52	0.32 ± 6.94	0.240

LV mass index, g/m^2^	B	79.60 ± 13.85	83.11 ± 12.80	83.65 ± 16.66∗	84.92 ± 12.33^†^	0.003
	F	79.71 ± 13.62	81.34 ± 12.32	83.23 ± 12.80	85.25 ± 13.44^‡^	0.001
	Δ	0.11 ± 11.24	−1.77 ± 10.43	−0.42 ± 11.32	0.34 ± 9.97	0.231

IVST, mm	B	8.51 ± 0.77	8.85 ± 0.94^‡^	9.04 ± 0.78^‡^	9.27 ± 0.86^‡^	<0.001
	F	8.62 ± 0.79	8.89 ± 0.92^‡^	9.04 ± 0.67^‡^	9.32 ± 0.91^‡^	<0.001
	Δ	0.11 ± 0.72	0.04 ± 0.75	0.00 ± 0.74	0.05 ± 0.67	0.381

LVPWT, mm	B	8.37 ± 0.72	8.63 ± 0.64^‡^	8.84 ± 0.67^‡^	9.03 ± 0.62^‡^	<0.001
	F	8.43 ± 0.67	8.64 ± 0.61^†^	8.85 ± 0.57^‡^	9.00 ± 0.78^‡^	<0.001
	Δ	0.06 ± 0.72	0.01 ± 0.62	0.01 ± 0.66	−0.03 ± 0.76	0.453

RWT	B	0.37 ± 0.03	0.37 ± 0.03	0.37 ± 0.02	0.37 ± 0.03	0.121
	F	0.38 ± 0.16	0.37 ± 0.03	0.37 ± 0.03	0.37 ± 0.03	0.208
	Δ	0.01 ± 0.16	0.00 ± 0.03	−0.00 ± 0.03	−0.00 ± 0.03	0.145

LVEF, %	B	67.44 ± 3.87	67.16 ± 3.30	66.71 ± 3.54	66.50 ± 3.29∗	0.008
	F	66.91 ± 3.31	66.64 ± 3.41	66.49 ± 3.19	66.27 ± 3.19	0.140
	*Δ*	−0.53 ± 4.37	−0.52 ± 4.34	−0.23 ± 3.98	−0.24 ± 3.79	0.708

E, cm/s	B	12.28 ± 2.49	11.94 ± 2.38	11.97 ± 2.43	11.80 ± 2.34	0.132
	F	11.06 ± 2.72	10.73 ± 2.73	10.39 ± 2.34∗	9.80 ± 2.29^‡^	<0.001
	Δ	−1.22 ± 2.53	−1.21 ± 2.20	−1.58 ± 2.40	−2.00 ± 2.30^‡^	<0.001

A, cm/s	B	66.54 ± 16.15	67.18 ± 17.18	65.85 ± 16.50	65.80 ± 15.79	0.737
	F	66.83 ± 16.18	66.38 ± 17.00	66.33 ± 16.61	66.05 ± 16.44	0.959
	Δ	0.34 ± 13.93	−0.84 ± 13.98	0.55 ± 13.40	0.05 ± 13.66	0.660

E/A	B	1.28 ± 0.39	1.21 ± 0.40	1.21 ± 0.36	1.19 ± 0.34	0.057
	F	1.26 ± 0.37	1.25 ± 0.41	1.22 ± 0.37	1.20 ± 0.34	0.211
	Δ	−0.02 ± 0.30	0.04 ± 0.33	0.00 ± 0.32	0.01 ± 0.32	0.138

e′ septal, cm/s	B	10.69 ± 2.35	10.41 ± 2.14	10.43 ± 2.24	10.26 ± 2.20	0.166
	F	9.43 ± 2.46	9.08 ± 2.37	8.90 ± 2.12∗	8.33 ± 2.10^‡^	<0.001
	Δ	−1.25 ± 2.56	−1.33 ± 2.22	−1.53 ± 2.39	−1.93 ± 2.25^†^	0.005

e′ lateral, cm/s	B	13.86 ± 3.04	13.47 ± 2.93	13.50 ± 2.94	13.34 ± 2.79	0.186
	F	12.68 ± 3.27	12.38 ± 3.33	11.88 ± 2.82∗	11.26 ± 2.76^‡^	<0.001
	Δ	−1.18 ± 3.03	−1.09 ± 2.63	−1.62 ± 2.84	−2.08 ± 2.76^†^	<0.001

E/e′ septal	B	7.81 ± 1.71	7.56 ± 1.79	7.50 ± 1.76	7.55 ± 1.79	0.179
	F	8.91 ± 2.20	8.93 ± 2.15	8.97 ± 2.09	9.53 ± 2.49^†^	0.003
	Δ	1.10 ± 2.35	1.37 ± 2.15	1.47 ± 2.14	1.97 ± 2.24^‡^	<0.001

E/e′ lateral	B	6.04 ± 1.42	5.87 ± 1.43	5.78 ± 1.48	5.88 ± 1.66	0.257
	F	6.64 ± 1.73	6.61 ± 1.78	6.78 ± 1.76	7.09 ± 1.95∗	0.009
	Δ	0.60 ± 1.82	0.75 ± 1.55	1.00 ± 1.79∗	1.21 ± 1.72^‡^	<0.001

E/e′ average	B	6.92 ± 1.46	6.71 ± 1.54	6.64 ± 1.54	6.72 ± 1.64	0.175
	F	7.78 ± 1.86	7.77 ± 1.86	7.87 ± 1.82	8.31 ± 2.11^†^	0.003
	Δ	0.85 ± 1.96	1.06 ± 1.72	1.23 ± 1.82	1.59 ± 1.83^‡^	<0.001

Data are expressed as mean ± standard deviation or median (interquartile range). ∗*P* < 0.05, ^†^*P* < 0.01, ^‡^*P* < 0.001. B: baseline; Δ: changes in corresponding parameters; F: follow-up; IVST: interventricular septal thickness; LA: left atrium; LV: left ventricle; LVEDD: left ventricular end-diastolic diameter; LVEDV: left ventricular end-diastolic volume; LVEF: left ventricular ejection fraction; LVESD: left ventricular end-systolic diameter; LVESV: left ventricular end-systolic volume; LVPWT: left ventricular posterior wall thickness; RWT: relative wall thickness; SUA: serum uric acid.

**Table 3:** Univariate and multivariate regression analysis for LVDD progression.

Variate	Univariate		Multivariate (SUA as continuous variable)		Multivariate (SUA as categorical variable)	
	OR (95% CI)	*P*	OR (95% CI)	*P*	OR (95% CI)	*P*
Age, per 10 y	2.466 (2.138~2.864)	<0.001	2.740 (2.330~3.249)	<0.001	2.839 (2.406~3.379)	<0.001
Male sex	1.908 (1.462~2.503)	<0.001	—	—	—	—
BMI, per kg/m^2^	1.027 (0.982~1.075)	0.242	—	—	—	—
Systolic BP, per 10 mmHg	1.232 (1.122~1.357)	<0.001	—	—	—	—
Diastolic BP, per 10 mmHg	1.221 (1.063~1.406)	0.005	1.143 (1.003~1.304)	0.046	1.147 (1.006~1.310)	0.041
Hypertension	2.638 (1.581~4.431)	<0.001				
Diabetes	2.595 (1.852~3.648)	<0.001	1.776 (1.079~2.939)	0.024	1.676 (1.016~2.783)	0.044
Smoking	1.461 (1.067~2.000)	0.018	—	—	—	—
HbA1c, per 1%	1.688 (1.380~2.088)	<0.001	0.771 (0.577~1.023)	0.073	0.792 (0.589~1.055)	0.114
Fasting glucose, per mmol/L	1.230 (1.105~1.381)	<0.001	—	—	—	—
Fasting insulin, per *μ*U/L	1.002 (0.992~1.013)	0.638	—	—	—	—
HOMA-IR, per unit	1.002 (0.973~1.027)	0.880	—	—	—	—
Triglyceride, per mmol/L	1.436 (1.255~1.659)	<0.001	1.440 (1.233~1.705)	<0.001	1.425 (1.219~1.690)	<0.001
Total cholesterol, per mmol/L	0.954 (0.844~1.077)	0.444	—	—	—	—
HDL cholesterol, per mmol/L	0.361 (0.243~0.530)	<0.001	—	—	—	—
LDL cholesterol, per mmol/L	0.911 (0.788~1.051)	0.202	—	—	—	—
eGFR, per 10 mL/min/1.732 m^2^	0.750 (0.685~0.818)	<0.001	—	—	—	—
LVEF, per 1%	0.942 (0.909~0.976)	<0.001	0.919 (0.881~0.959)	<0.001	0.917 (0.879~0.957)	<0.001
Beta blocker	1.889 (1.114~3.208)	0.018	—	—	—	—
ACEI/ARB	2.403 (1.380~4.239)	0.002	—	—	—	—
Calcium channel blocker	3.038 (1.489~6.465)	0.003	—	—	—	—
Statin	2.997 (1.872~4.869)	<0.001	—	—	—	—
Uric acid, per 100 *μ*mol/L	1.528 (1.311~1.786)	<0.001	1.351 (1.125~1.625)	0.001	/	/
Quartiles of uric acid, *μ*mol/L	—	—	—	—	—	—
≤282	Ref	—	—	—	Ref	—
283~340	1.171 (0.809~1.696)	0.403	—	—	0.753 (0.491~1.151)	0.190
341~ 393	1.376 (0.958~ 1.983)	0.085	—	—	0.893 (0.586~ 1.359)	0.596
≥ 394	2.697 (1.892~ 3.867)	<0.001	—	—	1.851 (1.215~ 2.828)	0.004

ACEI: angiotensin-converting enzyme inhibitor; ARB: angiotensin receptor blocker; BMI: body mass index; BP: blood pressure; eGFR: estimated glomerular filtration rate; HbA1c: glycated hemoglobin A1c; HDL: high-density lipoprotein; HOMA-IR: homeostasis model assessment-estimated insulin resistance; LDL: low-density lipoprotein; LVEF: left ventricular ejection fraction; SUA: serum uric acid.

## Data Availability

The datasets analyzed within the study are available from the corresponding author on reasonable request.

## Abbreviations

ALT: Alanine aminotransferase
AST: Aspartate aminotransferase
BMI: Body mass index
BSA: Body surface area
DtE: E wave deceleration time
eGFR: Estimated glomerular filtration rate
EF: Ejection fraction
HDL: High-density lipoprotein
HF: Heart failure
HFpEF: Heart failure with preserved ejection fraction
HOMA-IR: Homeostasis model assessment-estimated insulin resistance
hsCRP: High sensitive C-reactive protein
IVST: Interventricular septal thickness
LDL: Low-density lipoprotein
LV: Left ventricle
LVDD: Left ventricular diastolic dysfunction
LVEDD: Left ventricular end-diastolic diameter
LVEDV: Left ventricular end-diastolic volume
LVESV: Left ventricular end-systolic volume
LVPWT: Left ventricular posterior wall thickness
MetS: Metabolic syndrome
OGTT: Oral glucose tolerance test
PCA: Principal component analysis
RMFP: Restrictive mitral filling pattern
ROS: Reactive oxygen species
SUA: Serum uric acid
TDI: Tissue Doppler imaging
XO: Xanthine oxidase.

## Supplementary Materials

Supplementary Materials Supplementary Table I. Correlation analysis between cardiometabolic variables. Supplementary Table II. Factors analysis for LVDD progression.

## References

[1] Ponikowski P., Voors A. A., Anker S. D. (2016). 2016 ESC guidelines for the diagnosis and treatment of acute and chronic heart failure: the task force for the diagnosis and treatment of acute and chronic heart failure of the European Society of Cardiology (ESC) developed with the special contribution of the heart failure association (HFA) of the ESC. *European Heart Journal*.

[2] Yancy C. W., Jessup M., Bozkurt B. (2017). 2017 ACC/AHA/HFSA focused update of the 2013 ACCF/AHA guideline for the management of heart failure: a report of the American College of Cardiology/American Heart Association Task Force on Clinical Practice Guidelines and the Heart Failure Society of America. *Circulation*.

[3] Borlaug B. A. (2014). The pathophysiology of heart failure with preserved ejection fraction. *Nature Reviews. Cardiology*.

[4] Silbiger J. J. (2019). Pathophysiology and echocardiographic diagnosis of left ventricular diastolic dysfunction. *Journal of the American Society of Echocardiography: Official Publication of the American Society of Echocardiography.*.

[5] Abhayaratna W. P., Marwick T. H., Smith W. T., Becker N. G. (2006). Characteristics of left ventricular diastolic dysfunction in the community: an echocardiographic survey. *Heart*.

[6] Redfield M. M., Jacobsen S. J., Burnett J. C., Mahoney D. W., Bailey K. R., Rodeheffer R. J. (2003). Burden of systolic and diastolic ventricular dysfunction in the community: appreciating the scope of the heart failure epidemic. *Journal of the American Medical Association*.

[7] From A. M., Scott C. G., Chen H. H. (2010). The development of heart failure in patients with diabetes mellitus and pre- clinical diastolic dysfunction: a population-based study. *Journal of the American College of Cardiology*.

[8] Fischer M., Baessler A., Hense H. W. (2003). Prevalence of left ventricular diastolic dysfunction in the community. results from a Doppler echocardiographic-based survey of a population sample. *European Heart Journal*.

[9] Kuznetsova T., Herbots L., López B. (2009). Prevalence of left ventricular diastolic dysfunction in a general population. *Circulation Heart Failure*.

[10] Kane G. C., Karon B. L., Mahoney D. W. (2011). Progression of left ventricular diastolic dysfunction and risk of heart failure. *Journal of the American Medical Association*.

[11] Zhang Y., Safar M. E., Iaria P., Agnoletti D., Protogerou A. D., Blacher J. (2010). Prevalence and prognosis of left ventricular diastolic dysfunction in the elderly: the PROTEGER study. *American Heart Journal*.

[12] Boyer J. K., Thanigaraj S., Schechtman K. B., Perez J. E. (2004). Prevalence of ventricular diastolic dysfunction in asymptomatic, normotensive patients with diabetes mellitus. *The American Journal of Cardiology*.

[13] Poirier P., Bogaty P., Garneau C., Marois L., Dumesnil J. G. (2001). Diastolic dysfunction in normotensive men with well-controlled type 2 diabetes: importance of maneuvers in echocardiographic screening for preclinical diabetic cardiomyopathy. *Diabetes Care*.

[14] von Bibra H., St John Sutton M. (2010). Diastolic dysfunction in diabetes and the metabolic syndrome: promising potential for diagnosis and prognosis. *Diabetologia*.

[15] Dinh W., Lankisch M., Nickl W. (2011). Metabolic syndrome with or without diabetes contributes to left ventricular diastolic dysfunction. *Acta Cardiologica*.

[16] Russo C., Jin Z., Homma S. (2011). Effect of obesity and overweight on left ventricular diastolic function: a community-based study in an elderly cohort. *Journal of the American College of Cardiology*.

[17] Feig D. I., Kang D. H., Johnson R. J. (2008). Uric acid and cardiovascular risk. *The New England Journal of Medicine*.

[18] Glantzounis G. K., Tsimoyiannis E. C., Kappas A. M., Galaris D. A. (2005). Uric acid and oxidative stress. *Current Pharmaceutical Design*.

[19] Aroor A. R., Jia G., Habibi J. (2017). Uric acid promotes vascular stiffness, maladaptive inflammatory responses and proteinuria in western diet fed mice. *Metabolism*.

[20] Alcaino H., Greig D., Chiong M. (2008). Serum uric acid correlates with extracellular superoxide dismutase activity in patients with chronic heart failure. *European Journal of Heart Failure*.

[21] Patetsios P., Song M., Shutze W. P. (2001). Identification of uric acid and xanthine oxidase in atherosclerotic plaque. *The American Journal of Cardiology*.

[22] Georgiopoulos G., Tsioufis C., Kalos T. (2019). Serum uric acid is independently associated with diastolic dysfunction in apparently healthy subjects with essential hypertension. *Current Vascular Pharmacology*.

[23] Cicoira M., Zanolla L., Rossi A. (2002). Elevated serum uric acid levels are associated with diastolic dysfunction in patients with dilated cardiomyopathy. *American Heart Journal*.

[24] Sung K. T., Lo C. I., Lai Y. H. (2020). Associations of serum uric acid level and gout with cardiac structure, function and sex differences from large scale asymptomatic Asians. *PLoS One*.

[25] Alberti K. G., Zimmet P. Z., WHO Consultation (1998). Definition, diagnosis and classification of diabetes mellitus and its complications. Part 1: diagnosis and classification of diabetes mellitus provisional report of a WHO consultation. *Diabetic Medicine*.

[26] Chobanian A. V., Bakris G. L., Black H. R. (2003). Seventh report of the joint national committee on prevention, detection, evaluation, and treatment of high blood pressure. *Hypertension*.

[27] Yang C. D., Shen Y., Ding F. H. (2020). Visit-to-visit fasting plasma glucose variability is associated with left ventricular adverse remodeling in diabetic patients with STEMI. *Cardiovascular Diabetology*.

[28] Nagueh S. F., Smiseth O. A., Appleton C. P. (2016). Recommendations for the evaluation of left ventricular diastolic function by echocardiography: an update from the American Society of Echocardiography and the European Association of Cardiovascular Imaging. *Journal of the American Society of Echocardiography: Official Publication of the American Society of Echocardiography.*.

[29] Aljaroudi W., Alraies M. C., Halley C. (2012). Impact of progression of diastolic dysfunction on mortality in patients with normal ejection fraction. *Circulation*.

[30] Innelli P., Sanchez R., Marra F., Esposito R., Galderisi M. (2008). The impact of aging on left ventricular longitudinal function in healthy subjects: a pulsed tissue Doppler study. *European Journal of Echocardiography*.

[31] Nikitin N. P., Witte K. K., Thackray S. D., de Silva R., Clark A. L., Cleland J. G. (2003). Longitudinal ventricular function: normal values of atrioventricular annular and myocardial velocities measured with quantitative two-dimensional color Doppler tissue imaging. *Journal of the American Society of Echocardiography: Official Publication of the American Society of Echocardiography*.

[32] Mottram P. M., Haluska B. A., Leano R., Carlier S., Case C., Marwick T. H. (2005). Relation of arterial stiffness to diastolic dysfunction in hypertensive heart disease. *Heart*.

[33] Vasan R. S., Benjamin E. J., Levy D. (1995). Prevalence, clinical features and prognosis of diastolic heart failure: an epidemiologic perspective. *Journal of the American College of Cardiology*.

[34] Shapiro L. M., McKenna W. J. (1984). Left ventricular hypertrophy. Relation of structure to diastolic function in hypertension. *British Heart Journal*.

[35] Tu C. M., Tseng G. S., Liu C. W. (2020). Serum uric acid may be associated with left ventricular diastolic dysfunction in military individuals. *Military Medicine*.

[36] Rajesh M., Mukhopadhyay P., Bátkai S. (2009). Xanthine oxidase inhibitor allopurinol attenuates the development of diabetic cardiomyopathy. *Journal of Cellular and Molecular Medicine*.

[37] Baldus S., Müllerleile K., Chumley P. (2006). Inhibition of xanthine oxidase improves myocardial contractility in patients with ischemic cardiomyopathy. *Free Radical Biology & Medicine*.

[38] Cingolani H. E., Plastino J. A., Escudero E. M., Mangal B., Brown J., Pérez N. G. (2006). The effect of xanthine oxidase inhibition upon ejection fraction in heart failure patients: La Plata study. *Journal of Cardiac Failure*.

[39] Ogino K., Kato M., Furuse Y. (2010). Uric acid-lowering treatment with benzbromarone in patients with heart failure: a double-blind placebo-controlled crossover preliminary study. *Circulation. Heart Failure*.

