# Supplementary material for: Serum Uric Acid Is Associated with the Progression of Left Ventricular Diastolic Dysfunction in Apparently Healthy Subjects
Source: Dis Markers. 2022 Oct 15;2022:9927254. doi: 10.1155/2022/9927254 (PMC9588337; doi:10.1155/2022/9927254)
Supplement: Supplementary Materials — Supplementary Table I. Correlation analysis between cardiometabolic variables. Supplementary Table II. Factors analysis for LVDD progression. [file 9927254.f1.docx]

**Supplementary Table I. Correlation analysis between cardiometabolic variables**

|  | BMI | HbA1c | FPG | PPG | FPI | PPI | HOMA-IR | HDL-c | LDL-c | TC | TG | SUA |
| --- | --- | --- | --- | --- | --- | --- | --- | --- | --- | --- | --- | --- |
| BMI | 1 | 0.150^‡^ | 0.190^‡^ | 0.200^‡^ | 0.440^‡^ | 0.310^‡^ | 0.450^‡^ | -0.390^‡^ | -0.035 | -0.064 | 0.340^‡^ | 0.330^‡^ |
| HbA1c |  | 1 | 0.530^‡^ | 0.510^‡^ | 0.210^‡^ | 0.180^‡^ | 0.320^‡^ | -0.210^‡^ | 0.005 | 0.012 | 0.190^‡^ | 0.160^‡^ |
| FPG |  |  | 1 | 0.560^‡^ | 0.330^‡^ | 0.250^‡^ | 0.530^‡^ | -0.240^‡^ | -0.034 | -0.023 | 0.220^‡^ | 0.110^‡^ |
| PPG |  |  |  | 1 | 0.240^‡^ | 0.550^‡^ | 0.350^‡^ | -0.180^‡^ | -0.035 | 0.018 | 0.230^‡^ | 0.073 |
| FPI |  |  |  |  | 1 | 0.540^‡^ | 0.970^‡^ | -0.360^‡^ | -0.011 | 0.007 | 0.430^‡^ | 0.210^‡^ |
| PPI |  |  |  |  |  | 1 | 0.530^‡^ | -0.190^‡^ | 0.071 | 0.057 | 0.260^‡^ | 0.130^†^ |
| HOMA-IR |  |  |  |  |  |  | 1 | -0.380^‡^ | -0.017 | 0.004 | 0.440^‡^ | 0.220^‡^ |
| HDL-c |  |  |  |  |  |  |  | 1 | 0.062^*^ | 0.210^‡^ | -0.540^‡^ | -0.410^‡^ |
| LDL-c |  |  |  |  |  |  |  |  | 1 | 0.900^‡^ | 0.210^‡^ | 0.097^†^ |
| TC |  |  |  |  |  |  |  |  |  | 1 | 0.280^‡^ | 0.048 |
| TG |  |  |  |  |  |  |  |  |  |  | 1 | 0.380^‡^ |
| SUA |  |  |  |  |  |  |  |  |  |  |  | 1 |

Shown are Spearman’s correlation coefficient between different cardiometabolic parameters. ^*^*P*<0.05, ^†^*P*<0.01, ^‡^*P*<0.001.

BMI, body mass index; FPG, fasting plasma glucose; FPI, fasting plasma insulin; HbA1c, glycated hemoglobin; HDL-c, high-density lipoprotein cholesterol; HOMA-IR, homeostatic model assessment of insulin resistance; LDL-c, low-density lipoprotein cholesterol; PPG, postprandial plasma glucose (2 hours); PPI, postprandial plasma insulin (2 hours); SUA, serum uric acid; TC, total cholesterol; TG, triglyceride.

**Supplementary Table II. Factors analysis for LVDD progression**

| Parameter | Factor 1  (insulin resistance) | Factor 2 (cholesterol) | Factor 3  (SUA & HDL) |
| --- | --- | --- | --- |
| BMI | 0.208 | 0.097 | 0.187 |
| HbA1c | 0.329 | -0.181 | -0.403 |
| FPG | 0.393 | -0.143 | -0.375 |
| PPG | 0.180 | -0.112 | -0.225 |
| HOMA-IR | **0.477** | 0.109 | -0.100 |
| FPI | **0.432** | 0.156 | 0.010 |
| PPI | 0.163 | 0.029 | -0.024 |
| HDL-c | -0.298 | -0.114 | **-0.423** |
| LDL-c | -0.166 | **0.574** | -0.307 |
| TC | -0.126 | **0.621** | -0.318 |
| TG | 0.248 | 0.315 | 0.215 |
| SUA | 0.164 | 0.245 | **0.424** |
| % Variance explained | 27.9 | 19.0 | 14.7 |
| % Cumulative variance | 27.9 | 46.9 | 61.5 |

BMI, body mass index; FPG, fasting plasma glucose; FPI, fasting plasma insulin; HbA1c, glycated hemoglobin A1c; HDL-c, high-density lipoprotein cholesterol; HOMA-IR, homeostasis model assessment-estimated insulin resistance; LDL-c, low-density lipoprotein cholesterol; LVDD, left ventricular diastolic dysfunction; PPG, postprandial plasma glucose (2 hours); PPI, postprandial plasma insulin (2 hours); SUA, serum uric acid; TC, total cholesterol; TG, triglyceride.
